# TAS1R3 and TAS2R38 Polymorphisms Affect Sweet Taste Perception: An Observational Study on Healthy and Obese Subjects

**DOI:** 10.3390/nu14091711

**Published:** 2022-04-20

**Authors:** Monia Cecati, Arianna Vignini, Francesca Borroni, Sofia Pugnaloni, Sonila Alia, Jacopo Sabbatinelli, Giulia Nicolai, Marina Taus, Andrea Santarelli, Mara Fabri, Laura Mazzanti, Monica Emanuelli

**Affiliations:** 1 Department of Clinical Sciences, Università Politecnica delle Marche, 60126 Ancona, Italy; moniacecati@gmail.com (M.C.); s.pugnaloni@pm.univpm.it (S.P.); s.alia@pm.univpm.it (S.A.); j.sabbatinelli@pm.univpm.it (J.S.); andrea.santarelli@univpm.it (A.S.); l.mazzanti@univpm.it (L.M.); m.emanuelli@univpm.it (M.E.); 2 Research Center of Health Education and Health Promotion, Università Politecnica delle Marche, 60126 Ancona, Italy; 3 Department of Clinical Sciences, School of Specialization in Clinical Nutrition, Università Politecnica delle Marche, 60126 Ancona, Italy; francesca.borroni7@gmail.com; 4 Dietology and Clinical Nutrition, Azienda Ospedaliero Universitaria Ospedali Riuniti di Ancona, 60126 Ancona, Italy; g.nicolai@gmail.com (G.N.); marina.taus@ospedaliriuniti.marche.it (M.T.); 5 Department of Life and Environmental Sciences, Università Politecnica delle Marche, 60131 Ancona, Italy; m.fabri@univpm.it; 6 Fondazione Salesi, Ospedale G. Salesi, 60123 Ancona, Italy; 7 New York-Marche Structural Biology Center (NY-MaSBiC), Università Politecnica delle Marche, 60131 Ancona, Italy

**Keywords:** obesity, single nucleotide polymorphisms, taste receptors, eating behavior, taste identification

## Abstract

Background: The inter-individual differences in taste perception find a possible rationale in genetic variations. We verified whether the presence of four different single nucleotide polymorphisms (SNPs) in genes encoding for bitter (*TAS2R38*; 145G > C; 785T > C) and sweet (*TAS1R3*; −1572C > T; −1266C > T) taste receptors influenced the recognition of the basic tastes. Furthermore, we tested if the allelic distribution of such SNPs varied according to BMI and whether the associations between SNPs and taste recognition were influenced by the presence of overweight/obesity. Methods: DNA of 85 overweight/obese patients and 57 normal weight volunteers was used to investigate the SNPs. For the taste test, filter paper strips were applied. Each of the basic tastes (sweet, sour, salty, bitter) plus pure rapeseed oil, and water were tested. Results: Individuals carrying the AV/AV diplotype of the *TAS2R38* gene (A49P G/G and V262 T/T) were less sensitive to sweet taste recognition. These alterations remained significant after adjustment for gender and BMI. Moreover, a significant decrease in overall taste recognition associated with BMI and age was found. There was no significant difference in allelic distribution for the investigated polymorphisms between normal and overweight/obese patients. Conclusions: Our findings suggest that overall taste recognition depends on age and BMI. In the total population, the inter-individual ability to identify the sweet taste at different concentrations was related to the presence of at least one genetic variant for the bitter receptor gene but not to the BMI.

## 1. Introduction

Obesity represents an important worldwide health problem that, in the last decades, has reached epidemic proportions. Many health disorders are related to obesity: infertility, asthma, renal dysfunction, cardiovascular complications, cancer, diabetes, hepatic dysfunction are the most frequent diseases resulting from an increased BMI (Body Mass Index) [1]. Pineda et al. [2] forewarn that the Member States in the WHO European Region will not be able to reach the target to halt obesity at 2010 levels by 2025, as established during the World Health Assembly in 2013—cultural dynamics have increased the tendency for junk food and modified exercise habits.

Food preferences and dietary habits are guided by taste identification, meaning an ability to correctly identify a taste, and metabolic processes [3]. A growing bulk of scientific papers are focused on demonstrating a correlation between taste perception and variations in taste genes [4,5,6].

Taste cells are organized in taste buds distributed on the surface of the tongue, soft palate, pharynx, and the upper part of the esophagus [7]. Sweet, salty, sour, bitter, and umami are the five modalities of taste that can be detected by most mammals [8]. Among these, bitterness is probably the most exhaustively studied taste. In humans, bitter taste perception relies on the family of TAS2R genes [9] which includes 25 types of functional genes located on chromosomes 5, 7, and 12 [10]. The TAS2R38 gene is one of the most studied genes in the TAS2R gene family. TAS2R38 encodes a seven transmembrane G protein-coupled receptor [9] binding to the thiourea group present both in synthetic compounds (e.g., phenylthiocarbamide (PTC); 6-n-propylthiouracil (PROP)) and also in the goitrin, a natural compound found in food plants [11]. Three single nucleotide polymorphisms (SNPs) (145G > C; 785T > C; 886T > C) at the TAS2R38 gene result in three amino acid substitutions at residues P49A, A262V, and V296I. The Proline49–Alanine262–Valine296 (PAV) haplotype is reported as a supertaster genotype [12]. Conversely, the AVI amino acid haplotype is defined as a non–taster genotype [13].

Several studies have suggested that PROP supertasters have a higher sensitivity than non-tasters to various oral stimuli. Actually, the relationship between sweet-liking and PROP tasting are not casually related but may be explained by differences in orosensory sensitivity [14,15,16]. However, recent evidence suggested that TAS2R38 polymorphisms are insufficient to explain the differential bitter response to PTC and PROP and that other genes are also involved [12,17,18].

Reed et al. showed that modifying genes could influence the individual ability to taste PTC/PROP [17]. Lipchock et al. measured the PAV-TAS2R38 allele expression in heterozygous individuals (PAV/AVI) and demonstrated that PAV-TAS2R38 expression level is related to individual differences in bitter sensory perception [18].

The TAS1R gene family is mainly responsible for detecting sweet taste. TAS1R2 and TAS1R3 genes belong to the TAS1R gene family and are codified for two receptors involved in the perception of sweet taste. They are organized to form a heterodimer transmembrane protein [19] which allows humans to taste a variety of sweet substances [20]. Genetic variations in TAS1R3 promoter region have been deeply characterized. Several studies have tried to associate the human sucrose taste sensitivity with the two polymorphisms, −1572C > T and −1266C > T [21], but the results are conflicting [22].

Thus, the primary aim of the present study was to verify the association between the presence of SNPs in genes encoding for bitter (TAS2R38 rs713598 and rs1726866) and sweet (and TAS1R3 rs307355 and rs35744813) taste receptors and the ability of adult Caucasian individuals to recognize solutions of the six basic tastes, assessed using a psychophysical chemical taste test [23]. Furthermore, we aimed to verify if the allelic distribution of the investigated SNPs varied between normal weight and overweight/obese subjects—classified according to the BMI—and whether the associations between SNPs and taste recognition were influenced by the presence of being overweight, and obesity.

## 2. Materials and Methods

### 2.1. Study Population

In total, 85 overweight and obese subjects (BMI ≥ 25 kg/m^2^) were recruited between February and July 2019 from the Dietology and Clinical Nutrition Department at “Ospedali Riuniti” Academic Hospital, Ancona, Italy. They were recruited at first appointment, before being given a restricted diet.

In total, 57 healthy volunteers were (BMI < 25 kg/m^2^) selected in the same period among hospital healthcare professionals and their relatives. All enrolled subjects were Caucasian.

Healthy control (NW) subjects who participated in the study presented BMI < 25 kg/m^2^ while overweight/obese (OW/OB) subjects were included if their BMI > 25 kg/m^2^. Inclusion criteria specified that all participants (NW, OW, and OB) had no major medical illness (e.g., diabetes, heart disease, asthma, chronic rhinitis, and food allergies). In addition, smokers, alcohol addicted subjects, patients taking drugs that could alter taste identification, cancer patients, patients with endocrine-metabolic pathologies, patients with chronic oral diseases, women who were pregnant or lactating, were also excluded from the study. Sample size determination was based on our previous studies evaluating taste sensitivity with the same strip system in different cohorts [24,25,26]. Considering a mean 0.15 ± 0.20 difference in the proportion of correct answers for each type of taste stimulus between subjects who are homozygous for the common variant and subjects carrying at least one copy of the minor allele, 29 subjects per group would be required to detect a difference with 80% power and a 5% two-sided type I error rate. A sample size of 116 subjects, i.e., 58 NW and 58 OW/OB subjects, was deemed as appropriate. To ensure an acceptable frequency of the minor alleles in the entire cohort analysis, we planned to enroll at least 25 additional OW/OB subjects.

A semi-quantitative Food Frequency Questionnaire (FFQ) [27] was administered by trained nutritionists (FB and MT) to collect information on food intake. The FFQ consists of 248 items concerning the frequency of consumption and the portion sizes of foods and beverages commonly consumed in Italy. The obtained food data were entered into an appropriate software (WinFood, Medimatica, Martinsicuro, Italy) through which the percentages of carbohydrates, lipids, proteins, and fiber that the subject consumed, were calculated.

All enrolled subjects consumed a Mediterranean Diet. While for the NW subjects it consisted of 15% proteins, 30% lipids, 55% carbohydrates, and 30 g of fiber, OW/OB subjects were on a free diet consisting of 13% proteins, 35% lipids, 52% carbohydrates, and 22 g of fiber, which corresponded to their daily diet at enrolment.

Participants were given no food or drink for 30 min before sample collection, after which a buccal swab was taken by scraping the inside of the cheek using 3” cotton-tipped swabs from Puritan Medical Products Company (Guilford, ME, USA). Body weight was measured with a scale without shoes and wearing minimal clothes, to the nearest 0.01 kg and height was measured to the nearest 0.1 cm with a stadiometer (Seca, Hamburg, Germany) at enrolment, according to standardized procedures described elsewhere [28].

The current study was performed in adherence to the guidelines of the Declaration of Helsinki, after the protocol was approved by the Review Board of Università Politecnica delle Marche (Protocol 200446). Written informed consent was given by all subjects enrolled in the study, prior to the anthropometric parameter measurement and execution of the taste test.

### 2.2. Taste Test

The taste test, performed at the Dietology and Clinical Nutrition Department, is based on filter paper strips, as previously described [23,24,25,26], soaked with four substances (NaCl, citric acid, sucrose, quinine hydrochloride), each presented at 4 different concentrations, evoking the 4 basic taste qualities (salty, acid, sweet, bitter); in addition, pure rapeseed oil and water were administered, evoking fat taste [29] and neutral taste.

The concentrations used are shown in Table 1. Distilled water was used as solvent and taste solutions were freshly prepared on the morning of the testing session. Stimuli were applied to the left and right side of the protruded tongue, just posterior of the anterior third, with filter paper strips soaked in the different solutions. Before each filter paper strip application, participants were asked to wash their mouth with water. The taste presentations were randomized, and the stimulated side of the mouth was alternated, with a single trial for each combination of type of stimulus, concentration, and side of stimulation. A total of 36 stimuli—18 for each side of the mouth—were tested. Patients were asked to identify the taste from a list of eight descriptions, i.e., “sweet, sour, salty, bitter, oil, water, nothing, I don’t know”, according to a multiple forced-choice.

The overall taste recognition score was determined as ratio of correct answers to the total number of tested stimuli. “Nothing” and “I don’t know” responses were considered as incorrect. Ratios of correct answers were reported separately for each type of stimulation were appropriate.

### 2.3. DNA Isolation and Genotyping

After buccal collection, swabs were dried for 20 min under the laboratory fume hood and then stored at +4 °C before being processed.

Genomic DNA was extracted from buccal swabs by using “Saliva DNA isolation kit” (Norgen Biotek Corporation, Thorold, Ontario, Canada). DNA concentration was checked by UV absorption at 260 and 280 nm. We designed an allele-specific PCR reaction for genotyping all the investigated polymorphisms.

With this aim, we projected two doubly mismatched primers carrying mismatches in their last 3′ nucleotide. This mismatch is fundamental for PCR experiments to amplify specifically one of the two polymorphic variants characterizing the polymorphism. Briefly, for each sample, we arranged two PCR reactions in parallel, combining a common primer and one of the two mismatched primers to detect which kind of polymorphic base was present.

We amplified a region located upstream of the polymorphic locus by an additional primer as internal control to verify the PCR reaction in both mixtures. PCR products were electrophoresed on 2% agarose gels. Primers used in this work are shown in Table 2.

### 2.4. Statistical Analysis

Taste recognition was expressed as a ratio of correct answers after administrating the four different stimuli at 4 concentrations of each taste (i.e., 16 stimuli) plus fat and water for a total of 18 stimuli. The Kolmogorov–Smirnov goodness-of-fit test was used to determine whether clinical variables and taste recognition scores were normally distributed.

For each biallelic marker, allele frequencies were calculated from the genotypes in the patient (overweight/obese subjects) and control (normal weight subjects) groups using the Hardy–Weinberg equilibrium. Deviation from Hardy–Weinberg equilibrium was assessed using the χ^2^ test. The putative haplotype information for TAS2R38 was inferred using PHASE 2.1 software [30].

Results are expressed as Means ± SD. Student’s *t*-tests with Bonferroni correction for multiple comparisons were used to compare the differences in the number of correct answers between subjects who were homozygous for the most frequent allele and those who were heterozygous/homozygous for the less frequent allele, for all the polymorphisms studied. Multiple and one-way ANOVAs with Tukey’s post hoc comparisons were used to compare differences among TAS2R38 haplotypes. Taste recognition was analyzed for association with the presence of the investigated polymorphisms by one-way ANCOVA and multivariate regression analysis after adjustment for age, gender, and BMI. Data were analyzed using SPSS (Version 25.0. Armonk, NY, USA: IBM Corp). *p*-values < 0.05 were considered statistically significant.

## 3. Results

### 3.1. Subject Characteristics and SNP Genotyping

A total of 142 subjects (85 OW/OB subjects, 57 NW volunteers) were recruited for this study. The clinical characteristics of all study subjects in relation to TAS2R38 rs713598 and rs1726866 and TAS1R3 rs307355 and rs35744813 polymorphisms are presented in Table 3. After adjusting the significance level for multiple comparisons (*n* = 4, adjusted *p* = 0.0125), no deviations from the Hardy–Weinberg equilibrium were detected for the TAS2R38 SNPs (rs713598, *p* = 0.180; rs1726866, *p* = 0.022), while significant deviations due to heterozygote deficit were reported for the TAS1R3 SNPs (rs307355, *p* < 0.001; rs35744813, *p* < 0.001). The TAS2R38 haplotype reconstruction yielded four different haplotypes, i.e., PA (frequency, 0.34; SE, 0.01); PV (frequency, 0.17; SE, 0.01); AA (frequency, 0.24; SE, 0.01); AV (frequency, 0.25; SE, 0.01). The haplotype frequencies according to the study group are presented in Table 3.

### 3.2. Association between TAS2R38 SNPs and Taste Recognition

**Figure 1:**
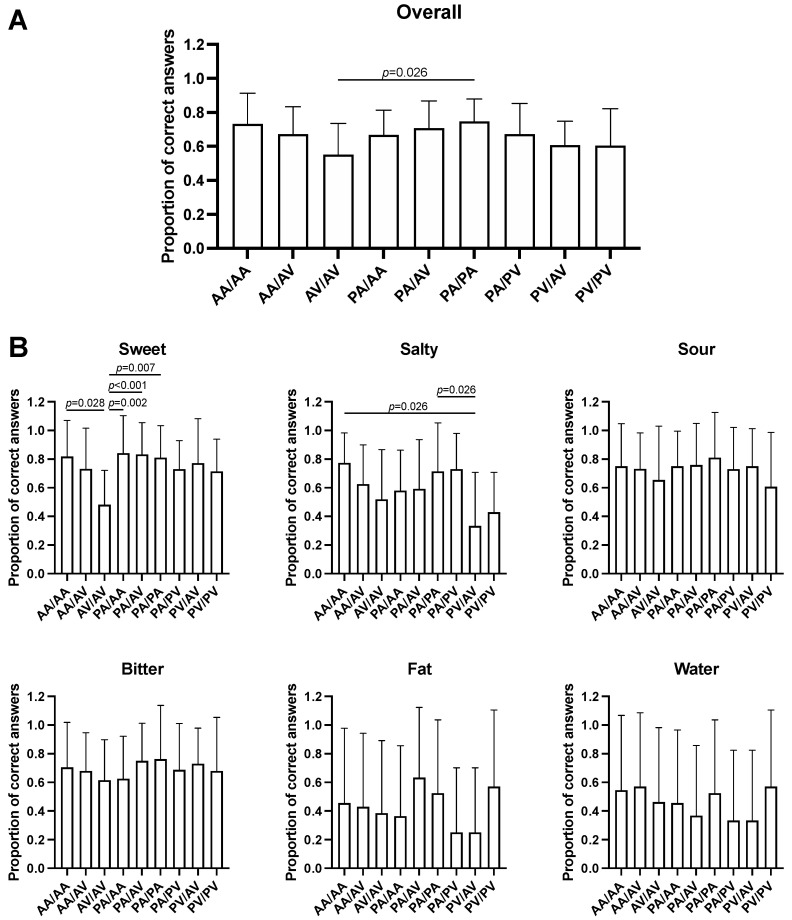
Evaluation of overall (**A**) and stimulus-specific (**B**) taste recognition for the TAS2R38 haplotypes. Data are represented as mean and standard deviations of the proportions of correct answers. *p* values from one-way ANOVA with Tukey’s post hoc test are shown.

Analysis of the taste recognition test results based on the TAS2R38 haplotypes revealed significant differences in overall taste recognition (F = 2.223, *p* = 0.029; Figure 1A), and in sweet (F = 2.981, *p* = 0.004; Figure 1B) and salty (F = 2.531, *p* = 0.013; Figure 1B) taste recognition among haplotypes. Multiple pairwise comparisons showed that the AV/AV diplotype is associated to a decreased sensitivity for the sweet taste compared to the AA/AA (mean difference, −0.38, *p* = 0.028), PA/AA (−0.36, *p* = 0.002), PA/AV (−0.35, *p* = 0.001), and PA/PA (−0.33, *p* = 0.007) diplotypes, whereas individuals carrying the PV/AV diplotype have a lower sensitivity for salty stimuli compared to those carrying the AA/AA (−0.44, *p* = 0.026) and PA/PA diplotypes (−0.38, *p* = 0.026). Moreover, individuals who carried the AV/AV diplotype reported a lower overall taste recognition compared to subjects who had the PA/PA diplotype (−0.20, *p* = 0.024).

### 3.3. Association between TAS1R3 SNPs and Taste Recognition

**Figure 2:**
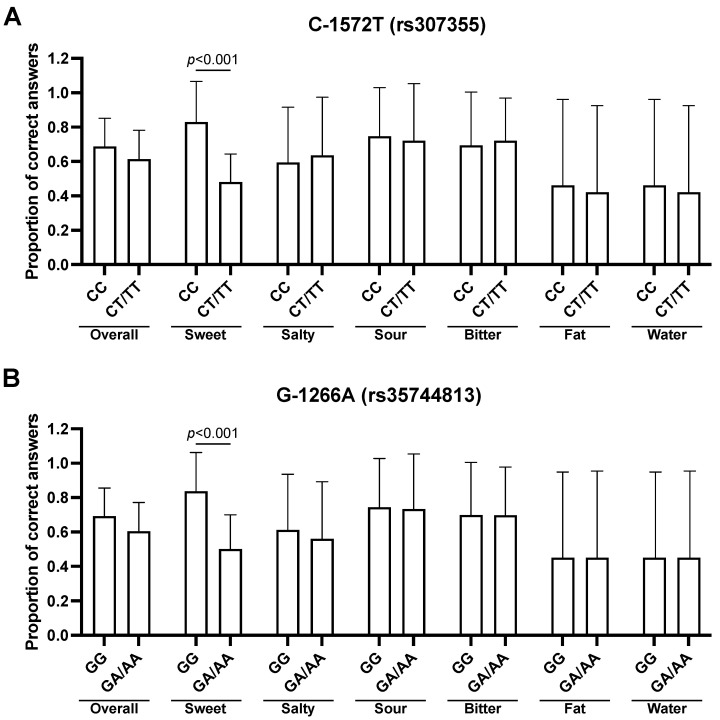
Evaluation of overall and stimulus-specific taste recognition for the (**A**) C-1572T and (**B**) G-1266A TAS1R3 SNPs. Data are represented as mean and standard deviations of the proportions of correct answers. Bonferroni-adjusted *p*-values for two-tailed *t*-test are reported.

Significant differences were found for sweet taste between the CC genotype and the CT/TT genotype (*rs307355*). These results suggest that subjects carrying the heterozygous or homozygous genotype for the T allele in the C-1572T SNP had a lower sensitivity for sweet taste (0.48 ± 0.16 vs. 0.83 ± 0.23, *t* = −6.906, adj. *p* < 0.001, Figure 2A). Significant differences were found also for sweet taste recognition between the G-1266A SNP GG genotype and the GA/AA genotypes. Subjects who have the homozygous genotype for the G allele in the G-1266A polymorphism have a higher sensitivity for sweet taste (0.84 ± 0.23 vs. 0.50 ± 0.20, *t* = −7.305, adj. *p* < 0.001, Figure 2B). In subjects carrying at least one of two polymorphisms for sweet, the difference remains confirmed (0.87 ± 0.20 vs. 0.50 ± 0.19, *t* = 9.806, adj. *p* < 0.001). No significant differences were found in overall taste recognition between subjects carrying or not carrying either SNP for the sweet taste receptor.

Statistical analysis revealed no significant differences in overall and specific taste recognition between heterozygous and homozygous subjects for all the four investigated SNPs (data not shown).

### 3.4. Effect of Overweight, Obesity, and Taste Receptor SNPs on Taste Recognition

The comparison between NW and overweight and obese subjects revealed a significant decrease in overall taste recognition (adj. *p* < 0.001), as well as in sensitivity to sour (adj. *p* = 0.022) stimuli in OW/OB subjects (Table 4). Moreover, a significant age-related decrease in the overall taste identification was reported (r = −0.267, *p* < 0.001) (data not shown).

There was no significant difference in allelic distribution of the four investigated polymorphisms between NW and OW/OB subjects (Table 3).

### 3.5. Multivariate Analysis for the Effects of TAS2R38 and TAS1R3 SNPs on Sweet Taste Recognition

To evaluate whether differences in sweet taste recognition remained significant even after adjustment for age-, sex-, and BMI, multiple multivariate linear regressions were computed for TAS2R38 and TAS1R3 receptors, first separately and then jointly.

The multivariate analysis showed that the TAS2R38 haplotype-related differences in the recognition of sweet stimuli observed at the univariate analysis were confirmed also in the age-, sex-, and BMI-adjusted multivariate model (Model 1; F(8,130) = 3.179, *p* = 0.003; Supplementary Table S1). Similarly, subjects carrying either one of the TAS1R3 C-1572T and G-1266A SNPs showed a lower taste recognition ability for sweet stimuli independent of age, sex, and BMI (Model 2; C-1572T, F(1,135) = 16.548, *p* < 0.001; G-1266A, F(1,135) = 13.265, *p* < 0.001; Supplementary Table S2).

A final multivariate analysis including all the SNPs under study was computed. After normalization by age, sex, and BMI, a significant decrease in sensitivity to sweet stimuli was found only in subjects carrying either one of the two SNPs for sweet taste receptor (Model 3; C-1572T, F(1,123) = 7.752, *p* = 0.006; G-1266A, F(1,123) = 7.915, *p* = 0.006) or the AV/AV TAS2R38 diplotype (F(13,128) = 9.040, *p* < 0.001; Table 5). Table 6 reports the adjusted estimated marginal means of taste recognition score for sweet stimuli according to the genotype for each multivariate model.

## 4. Discussion

The aim of the present study was to determine a possible association between SNPs in genes for bitter (TAS2R38 rs713598 and rs1726866) and sweet (and TAS1R3 rs307355 and rs35744813) taste receptors and measures of taste perception, in adult subjects of normal weight, overweight, and obese.

Here, we showed that subjects carrying the AV/AV TAS2R38 diplotype, which results from the combination of the A49P G/G and V262 T/T genotypes, have a reduced sweet, and overall, tasting ability, whereas individuals carrying the PV/AV TAS2R38 diplotype have a lower sensitivity for salty stimuli.

Several studies have focused their attention on the influence of AVI/PAV genotype on the sensitivity of subjects to sweet oral stimuli. Yeomans et al. reported an increased responsiveness of PROP supertasters to a wide range of oral stimuli including sweeteners [14]. Our study recognizes in a singular polymorphism of PAV/AVI genotype an increased importance to define the individual responsiveness to sweet oral stimuli: the reported data suggest, for the first time to our knowledge, that the AV/AV TAS2R38 diplotype can by itself decrease the perception of sweetness in investigated patients. However, no significant associations between taste sensitivity and food preferences were found (data not shown). Our data do not allow us to draw conclusions on the mechanisms linking TAS2R38 SNPs and sweet taste recognition. It is conceivable that bitter taste receptor SNPs could be co-inherited with other taste receptor SNPs which were not investigated in the present study, and therefore that the effect of TAS2R38 haplotypes on sweet taste responsiveness may be associative rather than causative. One of these hypotheses relies on the notion that proteins codified by TAS2R38 alleles have different capacities of binding to sucrose directly. This assumption also has some experimental support. Indeed, Pronin et al. [31] demonstrated that, within the proteins, the variant with V262A can bind sucrose.

Our results seem to suggest that the condition of being heterozygous for V262A polymorphism in the TAS2R38 gene could also be responsible for an improved perception of saltiness in the taster in agreement with data available in literature [14,15,16].

Regarding the sweet taste, our results suggest that subjects carrying the heterozygous or homozygous genotype for the T allele in the C-1572T SNP have a lower sensitivity for sweet taste. Moreover, subjects who have the homozygous genotype for the G allele in the G-1266A polymorphism, have a higher sensitivity for sweet taste. The observed difference remains unaltered when considering subjects carrying at least one of two polymorphisms for the sweet taste receptor. This latter consideration could find its possible rationale in the presence of other genes associated with the perception and the consumption of sweet substances. The TAS1R2 [32] and TAS1R3 [21] genes, a downstream gene GNAT3 [33], and recently the FTO [22] gene, have been shown to be related to the variance of sucrose sensitivity. According to Fushan et al. [21], we found that the heterozygous or homozygous genotype for the T allele in both investigated polymorphisms (rs307355 and rs35744813) is correlated with a lower sensitivity for sucrose in the subjects. Our data may find a possible explanation in a decreased translation of the TAS1R3 transmembrane protein since the presence of T allele is responsible for reduced promoter activity in comparison to the C allele [21].

Here, we confirmed previous evidence reporting that increasing BMI is associated with a reduction in the overall taste recognition score [26,34]. However, we found no significant difference in the SNP allele frequencies between the healthy volunteers and overweight/obese subjects. Moreover, our multivariate analysis showed that the effects of the investigated SNPs on the sweet taste recognition are independent of age, sex, and BMI. Hence, we can assume that the overweight/obese status does not influence the observed genotype-related differences in taste perception. Evidence for the association between obesity and the ability of recognizing specific tastes is still conflicting. Our results are in agreement with the report by Hwang et al. suggesting that increased BMI is associated with an alteration of taste perception of sweet taste perception [35]. On the other hand, a few studies found no direct association between BMI and sweet taste perception or sensitivity [36,37].

The generalizability of these results could be limited by the low allelic frequencies of the TAS1R3 SNPs. To address this issue, the regression models evaluating the impact of the four SNPs on the identification of sweet taste, were built on the entire study population while still considering the effects of BMI on taste recognition.

Nevertheless, there is no doubt that the present study could be improved by recruiting more patients and by analyzing other bitter receptor genes, bearing in mind that PROP perception could depend on more than the TAS2R38 gene [12].

In our opinion, this last evidence highlights, again, how research is still far from completely identifying the causes of a complex pathology such as obesity and how additional knowledge still needs to be acquired in the field of food preferences, choice and intake in relation to genetic factors including taste receptors of the gut.

However, further investigation is certainly needed to address this topic in a deeper and more thorough way.

## Figures and Tables

**Table 1:** Characteristics of taste stimuli.

Stimulus	Substance	Concentration (g/mL)
Sweet	Sucrose	−0.05
		−0.1
		−0.2
		−0.4
Salty	Sodium Chloride	−0.016
		−0.04
		−0.1
		−0.25
Bitter	Quinine hydrochloride	−0.0004
		−0.0009
		−0.0024
		−0.006
Sour	Citric acid	−0.05
		−0.09
		−0.165
		−0.3
Fat	Rape oil	Pure
Neutral	Deionized water	Pure

**Table 2:** Primer sets used for detecting polymorphic mutations. Red highlighted nucleotides are the mismatched nucleotides at the 3′ end of the primer.

SNP	Primer Fw (5’-3’)	Primer Rv (5’-3’)	Control Primer (5’-3’)
TAS2R38 A49P (rs713598)	GGTGGCAACCAGGTCTTTAG	CACAATCACTGTTGCTCAGTGC /CACAATCACTGTTGCTCAGTGG	GATGGCTTGGTAGCTGTGGT
TAS2R38 V262A (rs1726866)	CTTCTTTGTGATATCATCCTGTGT/ CTTCTTTGTGATATCATCCTGTGC	TGTGGTCGGCTCTTACCTTC	GGAAGGCACATGAGGACAAT
TAS1R3 C-1572T (rs307355)	AATGTGCAGGTGCCAGTTG	ACATGGTACACGCAAAGCG /ACATGGTACACGCAAAGCA	CACGGCACACACAATACACA
TAS1R3 G-1266A (rs35744813)	TGTGAGGGACACACACTACCA	ATGTATGCTGTGCACGTGC /ATGTATGCTGTGCACGTGT	GTGCCGTTTCCGTGTGTATT

**Table 3:** Clinical and demographic characteristics of all study subjects (*n* = 142) and allelic frequencies of the investigated SNPs.

	OW/OB (*n* = 85)	NW (*n* = 57)	*p*
BMI (Kg/m^2^)	31 ± 4.6	22 ± 2.3	<0.001
Gender (F/M)	52/33	41/16	0.186
Age (years)	45 ± 3	48 ± 4.2	0.857
Total Cholesterol (mg/dL)	194 ± 15	186 ± 13	0.081
HDL-Cholesterol (mg/dL)	41 ± 3	45 ± 4	0.072
LDL-Cholesterol (mg/dL)	124 ± 9	119 ± 8	0.068
Triglycerides (mg/dL)	141 ± 11	92 ± 7	0.061
TAS2R38 A49P-V262A haplotype frequencies			
PA (CC)	0.29	0.39	
PV (CT)	0.19	0.16	
AA (GC)	0.26	0.22	
AV (GT)	0.25	0.24	0.434
TAS1R3 C-1572T (rs307355) allelic frequencies			
C	0.86	0.94	
T	0.14	0.06	0.888
TAS1R3 G-1266A (rs35744813) allelic frequencies			
G	0.85	0.90	
A	0.15	0.10	0.370

**Table 4:** Comparison of the taste sensitivity between NW subjects and OW/OB patients. Data are mean (SD) of correct answer ratios. Bonferroni-adjusted *p*-values for two-tailed *t*-test are reported.

Stimuli	OW/OB (*n* = 85)	NW (*n* = 57)	*p*
Sweet	0.747 (0.278)	0.797 (0.227)	1
Salty	0.569 (0.311)	0.647 (0.341)	1
Bitter	0.658 (0.300)	0.759 (0.289)	0.336
Sour	0.682 (0.309)	0.828 (0.240)	0.022
Fat	0.310 (0.465)	0.660 (0.479)	<0.001
Neutral	0.390 (0.491)	0.530 (0.503)	0.709
Overall	0.629 (0.180)	0.740 (0.122)	<0.001

**Table 5:** Effects of age, BMI, sex, bitter, and sweet taste receptors polymorphism status on sweet taste recognition (ANCOVA, type III sum of squares; R^2^ = 0.573; adjusted R^2^ = 0.511).

Source of Variation	df	Mean Square	F	*p*	Partial eta Squared	Observed Power
Age	1	0.057	1.745	0.189	0.014	0.259
BMI	1	0.030	0.929	0.337	0.007	0.160
Gender	1	0.024	0.731	0.394	0.006	0.136
TAS2R38 haplotype	8	0.117	3.041	0.004	0.160	2.007
TAS1R3 C-1572T	1	0.254	7.752	0.006	0.059	0.789
TAS1R3 G-1266A	1	0.260	7.915	0.006	0.060	0.797
Error	128	0.039				

**Table 6:** Adjusted estimated marginal means of taste recognition to sweet stimuli for the investigated SNPs after adjustment for age, sex, and BMI. Data are mean and SEM for estimated marginal means. *p* values for two-tailed *t*-test and for post hoc analysis with Tukey’s correction are reported. SEM, standard error of the mean.

SNP	*n*	Adjusted Mean	SEM	*p*
Model 1—TAS2R38 diplotype				
AA/AA	11	0.861	0.076	Ref.
AA/AV	14	0.727	0.066	0.187
AV/AV	13	0.478	0.068	<0.001
PA/AA	22	0.852	0.052	0.928
PA/AV	30	0.842	0.046	0.837
PA/PA	21	0.790	0.056	0.458
PA/PV	12	0.757	0.072	0.315
PV/AV	12	0.794	0.072	0.518
PV/PV	7	0.730	0.094	0.275
Model 2—TAS1R3 SNPs				
TAS1R3 C-1572T (rs307355)				
CC	118	0.698	0.028	Ref.
CT/TT	24	0.490	0.042	0.001
TAS1R3 G-1266A (rs35744813)				
GG	113	0.685	0.034	Ref.
GA/AA	29	0.502	0.038	0.001
Model 3—Combined				
TAS2R38 diplotype				
AA/AA	11	0.683	0.0648	Ref.
AA/AV	14	0.608	0.0551	0.365
AV/AV	13	0.392	0.0560	< 0.001
PA/AA	22	0.690	0.0463	0.923
PA/AV	30	0.690	0.0414	0.918
PA/PA	21	0.662	0.0481	0.790
PA/PV	12	0.638	0.0598	0.590
PV/AV	12	0.642	0.0604	0.625
PV/PV	7	0.598	0.0780	0.381
TAS1R3 C-1572T (rs307355)				
CC	118	0.625	0.035	Ref.
CT/TT	24	0.465	0.046	0.006
TAS1R3 G-1266A (rs35744813)				
GG	113	0.626	0.039	Ref.
GA/AA	29	0.464	0.042	0.006

## Data Availability

The data sets generated and/or analyzed during the present study are available from the corresponding author on reasonable request.

## Supplementary Materials

The following supporting information can be downloaded at: https://www.mdpi.com/article/10.3390/nu14091711/s1, Table S1: Effects of age, BMI, sex and bitter taste receptor haplotype status on sweet taste recognition; Table S2: Effects of age, BMI, sex and sweet taste receptor polymorphism status on sweet taste recognition.
