# Supplementary material for: TAS1R3 and TAS2R38 Polymorphisms Affect Sweet Taste Perception: An Observational Study on Healthy and Obese Subjects
Source: Nutrients. 2022 Apr 20;14(9):1711. doi: 10.3390/nu14091711 (PMC9101038; doi:10.3390/nu14091711)
Supplement: Supplementary file 1 [file nutrients-14-01711-s001.zip › nutrients-1656354-supplementary.pdf]

**Supplementary Table S1.** Effects of age, BMI, sex and bitter taste receptor haplotype status on sweet taste recognition (ANCOVA, type III sum of squares; $R^2 = 0.185$; adjusted $R^2 = 0.117$).

| Source of variation | df | Mean square | F | p |
|---|---|---|---|---|
| Age | 1 | 0.204 | 3.441 | 0.066 |
| BMI | 1 | 0.008 | 0.138 | 0.711 |
| Gender | 1 | 0.068 | 1.155 | 0.285 |
| TAS2R38 diplotype | 8 | 0.188 | 3.179 | 0.003 |
| Error | 130 | 0.041 | | |

**Supplementary Table S2.** Effects of age, BMI, sex and sweet taste receptor polymorphism status on sweet taste recognition (ANCOVA, type III sum of squares; $R^2 = 0.419$; adjusted $R^2 = 0.393$).

| Source of variation | df | Mean square | F | p |
|---|---|---|---|---|
| Age | 1 | 0.048 | 1.185 | 0.278 |
| BMI | 1 | 0.045 | 1.098 | 0.297 |
| Gender | 1 | 0.009 | 0.226 | 0.635 |
| TAS1R3 C-1572T (rs307355, CC *vs* CT/TT) | 1 | 0.674 | 16.548 | <0.001 |
| TAS1R3 G-1266A (rs35744813, GG *vs* GA/AA) | 1 | 0.540 | 13.265 | <0.001 |
| Error | 135 | 0.041 | | |
